# Impact of a simplified treatment protocol for moderate acute malnutrition with a decentralized treatment approach in emergency settings of Niger

**DOI:** 10.3389/fnut.2023.1253545

**Published:** 2023-11-30

**Authors:** Luis Javier Sánchez-Martínez, Pilar Charle-Cuéllar, Abdoul Aziz Gado, Abdias Ogobara Dougnon, Atté Sanoussi, Nassirou Ousmane, Ramatoulaye Hamidou Lazoumar, Fanta Toure, Antonio Vargas, Candela Lucía Hernández, Noemí López-Ejeda

**Affiliations:** ^1^Unit of Physical Anthropology, Department of Biodiversity, Ecology and Evolution, Faculty of Biological Sciences, Complutense University of Madrid, Madrid, Spain; ^2^Action Against Hunger, Madrid, Spain; ^3^Action Against Hunger, Niamey, Niger; ^4^Action Against Hunger, West and Central Africa Regional Office, Dakar, Senegal; ^5^Nutrition Direction, Ministry of Health, Niamey, Niger; ^6^Centre de Recherche Médicale et Sanitaire (CERMES), Niamey, Niger; ^7^EPINUT Research Group (Ref. 920325), Faculty of Medicine, Complutense University of Madrid, Madrid, Spain

**Keywords:** wasting, simplified approaches, community health workers (CHWs), mid-upper arm circumference (MUAC), ready-to-use therapeutic food (RUTF), moderate wasting

## Abstract

**Introduction:**

Of the 45.4 million children under five affected by acute malnutrition in the world, the majority (31.8 million) are affected by moderate acute malnutrition (MAM). Its treatment is particularly complex in emergency settings such as the Diffa region in Niger. This study aims to evaluate the effectiveness and coverage of a simplified treatment protocol with Community Health Workers (CHWs) as treatment providers.

**Methods:**

This study is a non-randomized controlled trial. The control group (*n* = 181) received the standard protocol currently used in country, delivered by nursing staff only in health centres and health posts, while the intervention group (*n* = 483) received the simplified protocol which included nursing at health centres and CHWs at health post as treatment providers.

**Results:**

The recovery rate was higher in the simplified protocol group (99.6% vs. 79.56%, *p* < 0.001) recording lower time to recover and higher anthropometric gain. Treatment coverage in the intervention group increased from 28.8% to 84.9% and reduced in the control group (25.3% to 13.6%). No differences were found in the recovery rate of children treated by CHWs and nursing staff.

**Conclusion:**

The outcomes using the simplified protocol exceeded humanitarian requirements and demonstrated improvements compared to the standard protocol showing that the simplified protocol could be safely provided by CHWs in an emergency context. Further research in other contexts is needed to scale up this intervention.

## 1 Introduction

Globally, it is estimated that acute malnutrition currently affects 45.4 million children under the age of five. The West African region has one of the highest prevalence of global acute malnutrition (GAM) 6.9% [95% CI: (5.9%)–(8.1%)] on the African continent (1). Niger has one of the highest national rates of GAM in the region reaching 12.5% [95% CI: (11.1%)–(13.9%)] (2), which is above the 10% emergency threshold set by WHO / UNICEF indicating a high prevalence (3). The Diffa region, located in the southeast of the country, is particularly affected by the presence of chronic armed conflicts which negatively impacted food security, and has resulted in thousands of displaced people in the region who require humanitarian assistance (4).

National-level reports identify factors such as increased infant morbidity and mortality and its economic burden linked to medical treatments and loss of productivity as directly related to acute malnutrition prevalence in Niger (5). The term acute malnutrition refers to two well-known forms: severe acute malnutrition (SAM), the most extreme form which requires urgent treatment (6) due to the 9 times higher risk of death (7), and moderate acute malnutrition (MAM), precursor of SAM, with a 3 times higher risk of death compared to a well-nourished child (8), an increased risk of infectious diseases due to the deterioration of the immune system (7) but also longer term effects that affect the physical growth, cognitive development and future work capacity of the child (9). Recurrence of the condition is also a very important factor to consider as it has been shown that children who recover from MAM have high relapse rates in the year after their nutritional recovery (10).

The WHO officially recommends SAM and MAM to be treated independently following different protocols (11, 12), however there is a growing consensus that acute malnutrition should be considered as a continuous spectrum disorder, and not as two independent states (13). SAM is treated with ready-to-use therapeutic food (RUTF) in UNICEF-supplied outpatient therapeutic programmes (OTPs). Whereas MAM treatment programmes use supplementary foods, usually supported by the World Food Programme (WFP). Considerable discrepancies exist between the treatment protocols in different countries (14–18). MAM protocols may implement ready-to-use supplementary food (RUSF), fortified flours, nutrition education or counselling to improve child’s nutritional status (19, 20). Differences also exist in the cut-off points of the anthropometric indicators mid-upper arm circumference (MUAC) and/or weight-for-height Z-score (WHZ) that determine admission and cure. In some countries, guidelines for the treatment of MAM are absent (13).

Despite the implementation of Community-Based Management of Acute Malnutrition (CMAM) programming which allows children affected by severe or moderate acute malnutrition to be treated in outpatient settings, treatment coverage remains very low. In a review of 34 Supplementary Feeding Programmes, coverage was found to range from (10%) to 70% in rural settings, with a mean coverage of just 34.6%, and between 20% to 70% in urban settings with a mean coverage of 40.9% (21). Furthermore, in emergency situations, funding for SAM treatment programmes is commonly prioritised, leaving MAM children untreated (22).

In recent years, several simplified approaches have been developed to make treatment of acute malnutrition more accessible. Some of the main adaptations include using (i) Family MUAC for detection of cases, (ii) involving Community Health Workers (CHWs) as treatment providers, (iii) combined protocol (MUAC as the sole criterion for admission and recovery and using RUTF to treat both SAM and MAM cases), or (iv) reduced frequency of follow-up in specific contexts. These simplifications aim to identify cases earlier, increase coverage, facilitate their management, and reduce costs for governments (23). Several research studies have demonstrated the effectiveness of the two first approaches, mainly with SAM children (24–29). More evidence is needed related to the combined protocol and the potential impact of using different simplifications in the same context (30–34).

The aim of this study is to evaluate the effectiveness and coverage of MAM treatment in emergency settings in Niger using MUAC as the only criterion for admission and discharge, RUTF as nutritional product, delivered in both at health facilities and health post level.

## 2 Materials and methods

### 2.1 Treatment design and assessment

The study consisted of a non-randomized controlled trial conducted in nine communes in the Diffa region of Niger, between December 2020 and April 2021. The total sample of the study was 664 MAM children aged 6 to 59 months, who attended treatment centres spontaneously, or were recruited through active screening in the communities.

The experimental design comprised two groups receiving decentralized treatment in health posts but differing in terms of treatment provider and treatment protocol. The control group (*n* = 181), located in the catchment area of Kablewa, included its health centre and two health posts located in Kawa and Oudi Peulh villages. Children were treated following the standard CMAM protocol used in country with nursing staff as the only health care providers. The admission criteria of the control group consisted of WHZ < −2 and > −3 (35) and/or a MUAC<125 and > 115 mm and treatment was provided as a fixed dose of RUSF of 1 sachet/day (537 kcal/day, 12.1 g of protein, 35 g of lipids and 0 g of carbohydrates) (36). Discharge criteria were both WHZ > −2 and MUAC >125 mm during two consecutive visits.

The intervention group (*n* = 483) was located in N’Guigmi health area and comprised of its health centre and three health posts located in Birzoweya, Bonégrale and N’Gagala villages. Children were treated under a simplified protocol using a different nutritional product with both nurses and CHWs as treatment providers. In this group, the only admission criterion was MUAC <125 and > 115 mm, and treatment was provided with a fixed dose of RUTF of 1 sachet/day (500 kcal/day, 12.8 g of protein, 30.3 g of lipids and 45 g of carbohydrates) (36). The discharge criterion was MUAC >125 mm only, during two consecutive visits.

During admission the presence of comorbidities and type of admission (new, relapse, transfer…) were also recorded. The main variable considered in the study was treatment outcome which included: recovery (according to the anthropometric criteria outlined by each protocol), defaulting (children not showing up for the follow-up visit for two consecutive weeks), non-response (children that lost weight or with a stagnant weight gain for two consecutive visits) or discharge errors (children who appeared as cured on the records, but their anthropometric measures did not meet the criteria established by the protocol). Other outcome variables recorded included time to recovery, number of sachets used, and weight and MUAC gain.

### 2.2 Socio-economic assessment

To evaluate the possible socio-economic differences and their influence on treatment outcomes between the two groups, a socio-economic survey was carried out with a subsample of participants from each treatment arm (*n* = 107 treated with standard protocol and *n* = 296 treated with the simplified protocol). The survey was administered to the caregivers at the treatment sites and consisted of four groups of questions examining the dimensions of living conditions, namely demographics, livelihoods, food security and diversity assessed through the Food Consumption Score (37) and health care access.

### 2.3 Treatment coverage assessment

In addition to the main study, two coverage assessments were conducted, one prior to study enrolment in November 2020 and one at the end of the study in April 2021. Both assessments used the standardized methodology Simplified Lot Quality Assurance Sampling Evaluation of Access and Coverage (SLEAC) (38) in the same communes of the study and MAM cases were defined based on the anthropometric criteria described in the standard national CMAM protocol. In the first phase of this methodology a spatial sampling method was used to select the villages according to the distribution of the health centres. A survey was then conducted to find the number of current MAM cases registered in the programme (covered cases), the number of current MAM cases not registered in the programme (uncovered cases) and the number of recovering MAM children in the programme (did not have MAM at the time of the survey but had not yet been discharged as recovered).

### 2.4 Statistical analysis

Statistical analyses were performed using the R software (39). To assess the post-hoc statistical power, the sample size was calculated using the Fisher’s exact test for comparing two binomial proportions in two independent groups, under a 5% α error probability and a two-tail hypothesis of inequality (H_0_: p_1_ = p_2_ vs. H_1_: p_1_ ≠ p_2_) (40):


$$n={\left[\sqrt{\bar{p}\bar{q}\left(1+\frac{1}{k}\right)}\;z_{1-\alpha /2}+\sqrt{p_{1}q_{1}+\frac{p_{2}q_{2}}{k}z_{1-\beta }}\right]}^{2}/\Delta^{2}$$

where p_1_, p_2_ = projected true probabilities in the two groups. Estimated from previous studies (85% recovery for control group and 95% recovery for simplified group) (30, 41).

$Q_{1},q_{2}=1-p_{1},1-p_{2}$


$$\Delta =|p_{2}-p_{1}|$$

$$\bar{p}=\frac{p_{1}+kp_{2}}{1+k}$$

$$\bar{q}=1-\bar{p}$$

$$n={\left[1.96\cdot \sqrt{0.9\cdot 0.1\cdot \left(1+\frac{1}{1}\right)}+0.84\cdot \sqrt{0.85\cdot 0.15+\left(\frac{0.95\cdot 0.05}{1}\right)}\right]}^{2}/0.1^{2}=140$$

During data cleaning negative numbers or values greater than 4 standard deviations were considered as transcription errors resulting in 12 atypical values in the time to recovery and number of consumed sachets being eliminated. Normality of the quantitative variables was assessed through the Shapiro–Wilk test.

Univariate statistical comparisons between the two protocols were conducted using Pearson’s chi-square with the Yates continuity correction for the qualitative variables. Depending on the Normality of the distribution, Student’s t-test or Mann–Whitney test were used for the quantitative variables. For the coverage analysis, Mantel–Haenszel chi-square test (*p* < 0.05) was used to compare the final coverage of the treatment adjusted for the initial coverage in each of the study areas.

In the multivariate analyses the principal component analysis was applied to visualize the interdependence between the variables. The two-dimensional representation of treated children, using components 1 and 2, allowed the inclusion of the protocol variable in the graph that showed if there was a pattern related to the outcomes by using colours. Building on the dependency analysis results, different multiple linear regression models were conducted for each outcome to understand the effect of the included explanatory variables upon admission on treatment and their significance. To model the probability of cure over time, a multivariate Cox regression model was used to understand the associated Hazard Ratio (HR) of the explanatory variables included and adjusted for the impact of the others. Follow-up time, used in the analysis, was calculated from enrolment date to date of recovery. A Cox model describes the relation between the event incidence, as expressed by the hazard function and a set of covariates considering censored data (in our case the event is the recovery of treatment and the covariates considered in the model were: Protocol, sex, age, MUAC, comorbidities at admission and treatment provider). Mathematically, the Cox model is written as:

$h\left(t\right)=h_{0}\left(t\right)\times \exp\;\left\{b_{1}x_{1}+b_{2}x_{2}+\cdots +b_{p}x_{p}\right\}\text{.}$

where the hazard function h(t) is dependent on a set of p covariates (x_1_, x_2_, …, x_p_), whose impact is measured by the size of the respective coefficients (b_1_, b_2_, …, b_p_) (42). A forest plot was also used for the graphic representation of the results.

### 2.5 Ethical considerations

The study was approved by the National Health Research Ethics Committee of Niger, reference number 013/2020/CNERS. All parents or caretakers of the children included in the study signed informed consent prior participation in the study.

## 3 Results

The socioeconomic characteristics of the two study groups are presented in Table 1. No significant differences were found in relevant variables in terms of demographics, food security, or access to health. However, in the intervention group a lower proportion of participants was less than 30 min away from the health centre (36.0% vs. 51.4%, *p* = 0.008). The study groups presented more differences in terms of livelihoods, with the intervention group showing a higher proportion of families not owning a household nor arable land but, instead, a higher proportion of households with access to safe water, safe sanitation and electricity compared to the control group.

**Table 1:** Socioeconomic characteristics comparison between community management of acute malnutrition (CMAM) protocol group and the simplified protocol group.

Class	Indicator	Control CMAM protocol (*n* = 107)		Intervention simplified protocol (*n* = 296)		
		N° responses	Results	N° responses	Results	*p*-value
Demographics	Number of cohabiting people, mean (sd)	107	5.57 (2.59)	296	5.48 (2.24)	0.740
	Number of children under 5 years of age living with the treated child, mean (sd)	101	1.87 (1.95)	284	1.68 (1.75)	0.375
	Years of education of mother or primary caregiver, mean (sd)	69	0.77 (2.55)	173	0.60 (4.00)	0.748
Livelihoods	Type of housing	107		285		
	In propiety, % (*n*)		91.60 (98)		74.03 (211)	<0.001
	For rent, % (*n*)		0.00 (0)		7.02 (20)	0.011
	On loan, % (*n*)		8.40 (9)		18.95 (54)	0.017
	Households with access to safe water, % (*n*)	107	51.40 (55)	296	64.86 (192)	0.019
	Households with access to safe sanitation, % (*n*)	107	0.00 (0)	296	7.43 (22)	0.008
	Households with electricity, % (*n*)	107	4.67 (5)	296	13.51 (40)	0.021
	Households with arable land, % (*n*)	107	28.97 (31)	292	3.42 (10)	<0.001
	Households with livestock, % (*n*)	107	56.07 (60)	296	59.46 (176)	0.621
	N° cows, mean (sd)	60	6.28 (8.36)	176	6.94 (13.50)	0.657
	N° sheep, mean (sd)	60	3.88 (4.97)	176	1.42 (3.20)	<0.001
	N° goats, mean (sd)	60	8.13 (14.55)	176	7.73 (12.75)	0.848
	Households with construction land (concrete, cement, wood, tiles…), % (*n*)	107	0.93 (1)	287	0.70 (2)	0.999
	Households with construction roof (concrete, cement, wood, tiles…), % (*n*)	107	1.87 (2)	295	0.68 (5)	0.999
Food security	Number of meals per day, mean (sd)	102	2.93 (0.47)	268	2.77 (0.44)	0.003
	Lack of food in the last 4 weeks	107		291		
	No, % (*n*)		77.60 (83)		83.50 (243)	0.224
	Rarely, % (*n*)		22.40 (24)		15.45 (45)	0.139
	3–10 times, % (*n*)		0.00 (0)		0.70 (2)	0.952
	More than 10 times, % (*n*)		0.00 (0)		0.35 (1)	0.999
	Food Diversity (Food Consumption Score), mean (sd)	107	56.30 (21.16)	296	53.52 (19.60)	0.237
	Poor diet, % (*n*)		6.54 (7)		3.04 (9)	0.193
	Limited diet, % (*n*)		14.95 (16)		11.82 (35)	0.506
	Acceptable diet, % (*n*)		78.50 (84)		85.14 (252)	0.153
Heath care access	Behavior if the child is sick	106		296		
	Health centre or health post, % (*n*)		100.00 (106)		94.94 (281)	0.039
	Traditional medicine, % (*n*)		0.00 (0)		4.39 (13)	0.061
	Self medication, % (*n*)		0.00 (0)		0.67 (2)	0.965
	Households with difficulty to access treatment, % (*n*)	107	4.70 (5)	296	4.39 (13)	0.999
	Time it takes to get to treatment	105		294		
	30 min or less, % (*n*)		51.42 (54)		36.05 (106)	0.008
	Up to 1 h 30 min, % (*n*)		34.29 (36)		40.82 (120)	0.288
	More than 2 h, % (*n*)		14.29 (15)		23.13 (68)	0.076

CMAM: community management of acute malnutrition; sd: standard deviation; *n*: number of individuals.

**Figure 1:**
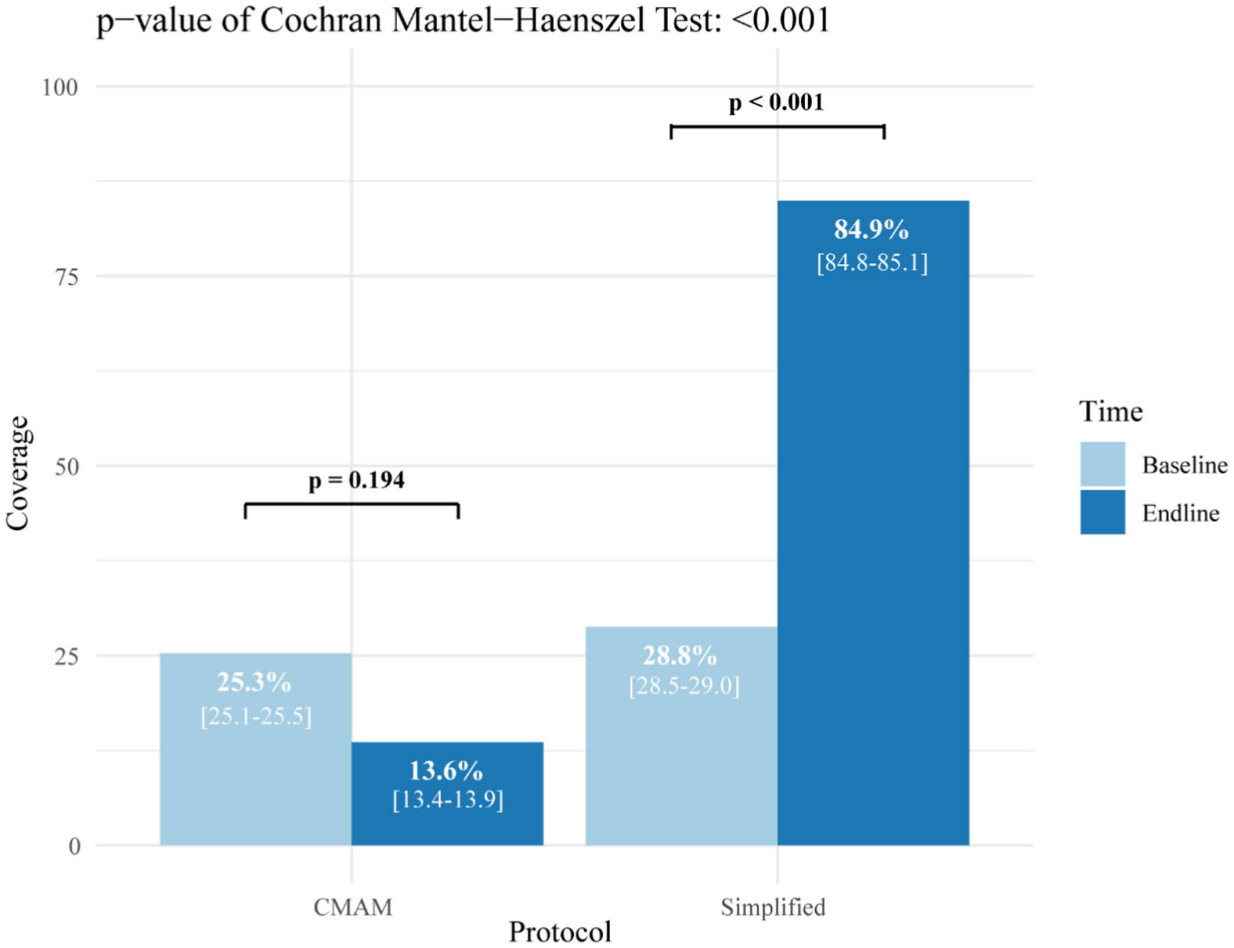
Coverage of moderate acute malnutrition treatment in the study groups before and after the intervention. CMAM: community management of acute malnutrition.

The treatment coverage estimates recorded at the beginning and end of the intervention are presented in Figure 1. During the study period, the intervention group registered an increase of 56% in treatment coverage while the control group recorded a 12% decrease. After adjusting for initial coverage, there was a significant difference in the final coverage between the intervention group (84.9%) and the control group (13.6%).

Table 2 summarizes the characteristics of children treated for malnutrition at the time of admission. In both groups the majority of cases were new admissions, however, children in the intervention group had significantly more cases of diarrhea, cough, fever, and pale conjunctiva than the control group. Significant differences were found for WAZ and MUAC between the groups, although the average difference was too small to be of clinical relevance, especially in the case of MUAC.

**Table 2:** Admission characteristics of children treated for moderate acute malnutrition by study group.

Characteristics	Control CMAM protocol	Intervention simplified protocol	Comparison
			*p*-value
Sex and age	*n* = 181	*n* = 483	
Female, *n* (%)	92 (50.83)	258 (53.42)	0.611^NS^
Age in months, mean (sd)	12.33 (4.47)	14.05 (6.09)	0.001
Presence of comorbidities	*n* = 181	*n* = 483	
Diarrheal, *n* (%)	2 (1.10)	48 (9.94)	<0.001
Vomiting, *n* (%)	2 (1.10)	6 (1.24)	0.999^NS^
Acute respiratory infection, *n* (%)	0 (0.00)	9 (1.86)	0.141^NS^
Cough, *n* (%)	4 (2.21)	59 (12.21)	<0.001
Fever, *n* (%)	2 (1.10)	25 (5.18)	0.032
Pale Conjunctiva, *n* (%)	0 (0.00)	27 (5.59)	0.002
Malaria positive test result, *n* (%)	0 (0.00)	0 (0.00)	0.999^NS^
Type of admission	*n* = 179	*n* = 476	
New admission, *n* (%)	179 (100)	472 (99.16)	0.504^NS^
Readmission, *n* (%)	0 (0.00)	0 (0.00)	0.999^NS^
Relapse, *n* (%)	0 (0.00)	3 (0.63)	0.678^NS^
Transfer, *n* (%)	0 (0.00)	1 (0.21)	0.999^NS^
Anthropometry at admission	*n* = 181	*n* = 481	
WHZ, mean (sd)	−2.18 (0.75)	−2.13 (1.17)	0.326^NS^
HAZ, mean (sd)	−1.45 (1.69)	−1.19 (1.56)	0.119^NS^
WAZ, mean (sd)	−2.37 (0.88)	−2.18 (0.91)	0.009
MUAC, mean (sd)	119.52 (1.88)	119.08 (2.03)	0.016

CMAM: community management of acute malnutrition; HAZ: height-for-age z-score; MUAC: middle-upper arm circumference; NS: Not significant *p*-value; sd: standard deviation; WAZ: weight-for-age z-score; WHZ: weight-for-height z-score; *n*: number of individuals.

Table 3 shows the results for anthropometric severity at admission by protocol group and treatment provider. Significant differences were found between treatment providers in simplified protocol in terms of median values of HAZ and WAZ but not for MUAC or WHZ, which are the outcome indicators with clinical relevance for acute malnutrition.

**Table 3:** Anthropometry at admission between study groups and treatment providers.

Protocol	CMAM protocol	Simplified protocol		
	*n* = 181	*n* = 481		
Provider	Health staff (in health centre and health post)	CHWs (in health posts)	Health staff (in health centre)	*p*-value
	*n* = 181	*n* = 208	*n* = 273	
WHZ, mean (sd)	−2.180 (0.748)	−2.185 (1.121)	−2.090 (1.208)	0.112
HAZ, mean (sd)	−1.451 (1.690)	−1.423 (1.390)	−1.015 (1.664)	0.002
WAZ, mean (sd)	−2.366 (0.882)	−2.327 (0.943)	−2.070 (0.863)	<0.001
MUAC, mean (sd)	119.508 (1.529)	119.177 (1.868)	119.014 (2.037)	0.184

CMAM: community management of acute malnutrition; CHWs: community health workers; WHZ: weight-for-height z-score; HAZ: height-for-age z-score; WAZ: weight-for-age z-score; MUAC: middle-upper arm circumference; sd: standard deviation; *n*: number of individuals.

The treatment outcomes of the groups are presented in Table 4. The proportion of children cured was 20% higher in the simplified protocol group with a post-hoc calculated power of 1.000 and an alpha error of 0.010. The same group presented fewer cases of defaulting, non-response, and discharge errors compared to the standard CMAM protocol. No deaths were recorded in any of the groups. The average time to recovery was two weeks shorter for children treated with the simplified protocol, registering a higher daily gain in both weight and MUAC, compared to the standard protocol despite using stricter discharge criteria.

**Table 4:** Treatment outcomes comparison among children treated for moderate acute malnutrition by study group.

Treatment outcomes	Control CMAM protocol	Intervention simplified protocol	
	*n* = 181	*n* = 483	*p*-value
Recovery, *n* (%)	144 (79.56)	481 (99.60)	<0.001
Death, *n* (%)	0 (0.00)	0 (0.00)	–
Defaulting, *n* (%)	8 (4.42)	0 (0.00)	<0.001
Non-response, *n* (%)	18 (9.94)	1 (0.20)	<0.001
Discharge error, *n* (%)*	11 (6.08)	1 (0.20)	<0.001
Time to recovery (days)** median (IQR)	*n* = 141	*n* = 472	<0.001
	42.00 (34.00; 56.00)	28.00 (21.00; 35.00)	
Food consumption** median (IQR)	*n* = 139	*n* = 474	
RUSF sachets	60.00 (45.00; 60.00)		–
RUTF sachets		28.00 (28.00; 35.00)	
Weight gain** median (IQR)	*n* = 139	*n* = 471	
Total (Kg)	1.00 (0.60; 1.30)	0.80 (0.50; 1.20)	0.010
Daily (g/Kg/day)	3.08 (2.18; 4.52)	4.46 (2.86; 6.85)	<0.001
MUAC gain** median (IQR)	*n* = 141	*n* = 472	
Total (mm)	8.00 (6.00; 10.00)	10.00 (8.00; 11.00)	<0.001
Daily (mm/day)	0.19 (0.14; 0.25)	0.34 (0.27; 0.48)	<0.001

CMAM: community management of acute malnutrition; IQR: interquartile range; MUAC: middle-upper arm circumference; RUSF: ready-to-use supplementary food; RUTF: ready-to-use therapeutic food; *n*: number of individuals. *Cases discharged as cured but whose anthropometry does not meet the cure criteria according to each protocol. **Variables recorded only among recovered children.

A comparison of outcomes between service providers was made within the intervention group (Table 5). Children treated by CHWs recovered on average 7 days earlier, consumed less therapeutic food, had higher daily weight gain which was almost double compared to children treated by health staff.

**Table 5:** Outcomes among recovered children treated of moderate acute malnutrition with the simplified protocol compared by treatment provider within the intervention group.

Treatment outcomes	Community health workers	Health staff	
	*n* = 208	*n* = 275	*p*-value
Recovery, *n* (%)	207 (99.52)	274 (99.64)	0.999
**Time to recovery (days)**	*n* = 203	*n* = 271	<0.001
	21.00 (21.00; 28.00)	28.00 (21.00; 36.00)	
**Food consumption median (IQR)**	*n* = 203	*n* = 273	
RUTF sachets	28.00 (28.00; 28.00)	35.00 (28.00; 42.00)	<0.001
**Weight gain median (IQR)**	*n* = 201	*n* = 270	
Total (Kg)	1.10 (0.67: 1.20)	0.70 (0.50; 1.00)	<0.001
Daily (g/Kg/day)	6.57 (3.69; 7.94)	3.57 (2.44; 5.02)	<0.001
MUAC gain** median (IQR)	*n* = 202	*n* = 270	
Total (mm)	10.00 (8.00; 12.00)	9.00 (8.00; 11.00)	<0.001
Daily (mm/day)	0.46 (0.33; 0.52)	0.30 (0.23; 0.37)	<0.001

MUAC: middle-upper arm circumference; IQR: interquartile range; RUSF: ready-to-use supplementary food; RUTF: ready-to-use therapeutic food; *n*: number of individuals.

Table 6 shows the variables that influence outcome indicators and the treatment protocol of children that achieved recovery. After adjusting for explanatory variables, it was found that treatment protocol, significantly affects treatment outcomes by reducing the consumption of food product and time of recovery while increasing the daily gain of MUAC and weight. Sex did not appear to have an influence on the outcome variables and age showed an association only with daily weight gain, which was lower the older was the child. Daily weight gain was significantly influenced by all explanatory variables except sex, while the daily MUAC gain was associated only to the initial MUAC value, the treatment protocol and its provider. The study also showed a significant association between lower MUAC values at admission and increased consumption of food products, and longer time to recovery. However, no significant association was found with WHZ, HAZ or WAZ at admission.

**Table 6:** Association of sex, age, admission characteristics and protocol in treatment variables of children cured from moderate acute malnutrition.

Dependent variable	Model 1: sachets consumption		Model 2: time to recovery (days)		Model 3: daily MUAC gain		Model 4: daily weight gain	
Independent variables	β coefficient (95% CI)	*p*-value	β coefficient (95% CI)	*p*-value	β coefficient (95% CI)	*p*-value	β coefficient (95% CI)	*p*-value
(Intercept)	270.27 (210.05; 330.49)	<0.001	301.11 (238.85; 363.38)	<0.001	1.86 (1.14; 2.58)	<0.001	−20.45 (−26.29; 13.04)	0.011
Sex: male	−0.44 (−2.43; 1.54)	0.660	−1.50 (−3.56; 0.56)	0.154	0.02 (−0.01; 0.04)	0.172	−0.38 (−1.66; −0.28)	0.153
Age	−0.12 (−0.32; 0.08)	0.242	−0.15 (−0.37; 0.06)	0.152	0.01 (−0.01; 0.01)	0.230	−0.08 (−0.18; −0.04)	0.004
Comorbidities: yes	1.76 (−1.12; 4.65)	0.230	1.05 (−1.89; 3.99)	0.483	−0.02 (−0.05; 0.01)	0.193	−1.08 (−2.22; −0.20)	0.004
MUAC	−1.77 (−2.27; −1.27)	<0.001	−2.11 (−2.63; −1.59)	<0.001	−0.01 (−0.02; −0.01)	<0.001	0.19 (−0.09; 0.24)	0.005
WHZ	−1.11 (−7.32; 5.10)	0.725	−6.57 (−13.13; −0.02)	0.049	0.02 (−0.05; 0.10)	0.587	2.18 (−0.49; 3.89)	0.010
HAZ	−0.36 (−4.85; 4.12)	0.874	−4.14 (−8.88; 0.64)	0.087	0.01 (−0.05; 0.06)	0.852	1.72 (0.04; 3.22)	0.005
WAZ	1.21 (−7.51; 9.93)	0.786	8.70 (−0.48; 17.89)	0.063	−0.01 (−0.11; 0.10)	0.857	−4.28 (−7.11; −0.97)	<0.001
Provider: CHWs	−6.20 (−8.36; −4.04)	<0.001	−6.83 (−9.06; −4.59)	<0.001	0.12 (0.09; 0.15)	<0.001	2.09 (1.61; 3.10)	<0.001
Protocol: simplified	−22.03 (−24.52; −19.54)	<0.001	−15.59 (−18.17; −13.01)	<0.001	0.10 (0.07; 0.13)	<0.001	0.81 (−0.44; 1.26)	0.016
Adjusted *R*^2^	0.47		0.36		0.29		0.19	

CHWs: community health workers; CI: confidence interval; HAZ: height-for-age z-score at admission; MUAC: middle-upper arm circumference at admission; WAZ: weight-for-age z-score at admission; WHZ: weight-for-height z-score at admission.

**Figure 2:**
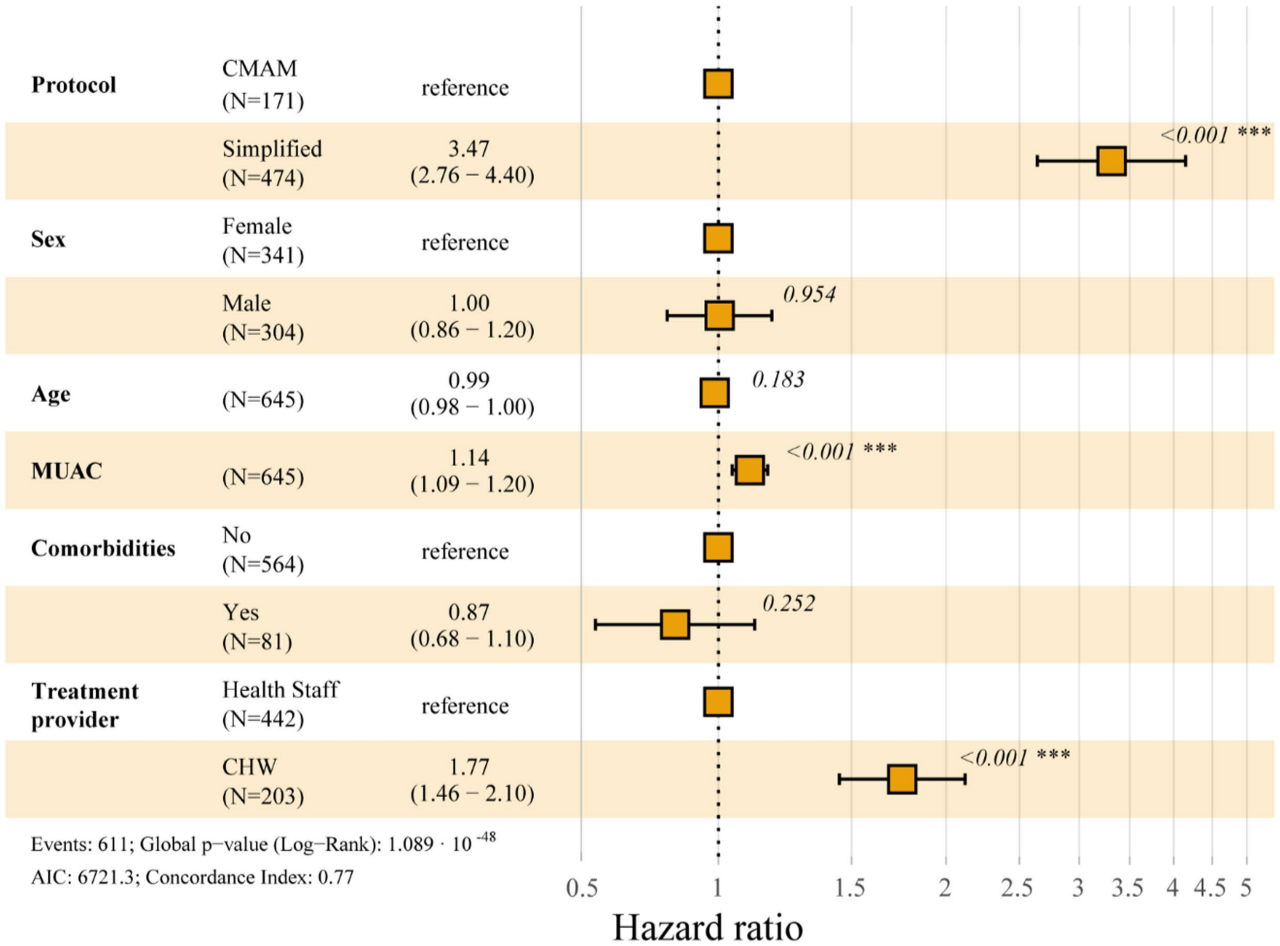
Forest plot displaying multivariate cox regression model of the probability of cure over time. CMAM: community management of acute malnutrition; MUAC: middle-upper arm circumference at admission; CHW: community health workers; N: number of individuals.

The results of the Cox regression analysis are presented in a Forest plot in Figure 2. A hazard ratio (HR) value of 1 indicates that the variable in question does not have an impact on the probability of recovery over time and, are not significant for recovery. The factor with the greatest significance was the treatment protocol, with the simplified protocol increasing the probability of child’s recovery by more than three times compared to the standard CMAM protocol. Being treated by a CHW compared to a health staff increased the probability of recovery by 77%. Lastly, every additional millimeter of MUAC presented at admission, increased the probability of recovery by 10%.

**Figure 3:**
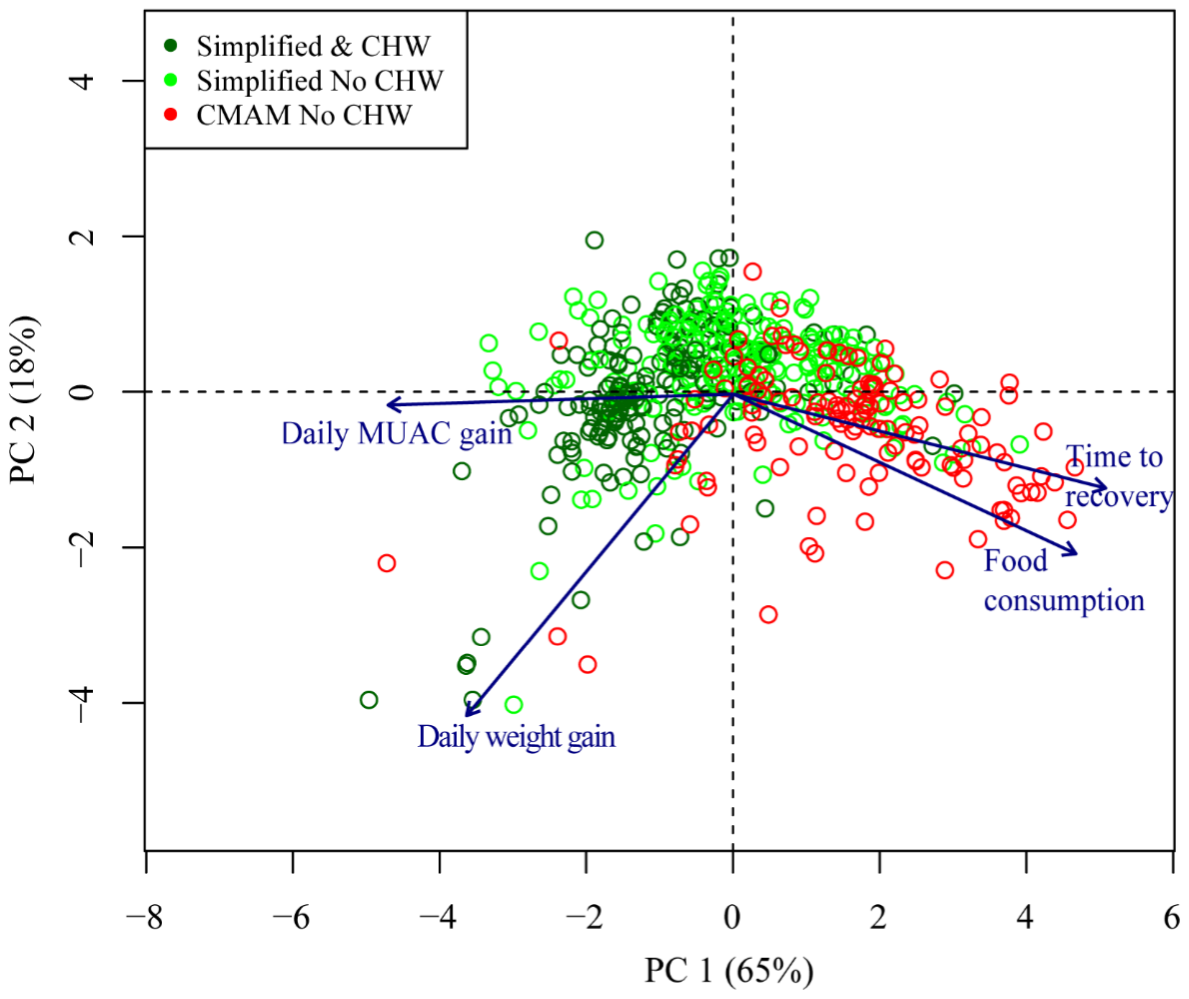
Principal component analysis showing the relationship between different quantitative results of interest and the protocol with which the patient has been treated. CMAM: community management of acute malnutrition; CHW: community health workers; MUAC: middle-upper arm circumference at admission; PC: principal component.

Figure 3 presents the principal component analysis (PCA) of the treatment outcomes in relation to the cured children in each study group and the treatment provider. Each child is represented by a sphere and its location within the quadrants depends on the combined effect of all the treatment variables included. Each independent variable is represented by an arrow showing the effect on the outcome, hence, the greater the influence, the more pronounced the displacement of a case will be in that direction. As expected, time to recovery and the total consumption of food product had a similar effect and were more influential for those cases treated with the standard CMAM protocol (in red). The anthropometric gain outcomes were instead associated with the cases treated with the simplified protocol (in green). Moreover, cases treated by CHWs (in dark green) were located more to the left, showing a stronger association with weight gain than MUAC gain, due to their diagonal dispersion.

## 4 Discussion

The present study showed that, in the emergency context of the Diffa region in Niger, the simplified protocol was able to cure more children, in less time, with greater anthropometric gain and less ready-to-use nutritional product use than the standard CMAM protocol.

The two study groups had similar socioeconomic characteristics, but differed in certain aspects, some having a positive effect on the intervention group and others on the control group, although neither group had a clear baseline disadvantage that could considerably influence treatment outcomes. No significant differences were found in anthropometric measurements; however, the simplified protocol group presented a greater number of cases with diarrhea, cough, fever, and pale conjunctiva, which could complicate recovery (43). Despite of this, the intervention group recorded better treatment outcomes with higher recovery rate and lower proportion of non-response.

After adjusting for baseline treatment coverage, the study showed a significant increase in coverage in the intervention group (+56.1%), and a decrease in the control group (−11.7%) demonstrating the influence of programme adaptations. While the simplified protocol group exceeded the 50% Sphere coverage standard for rural areas (44), the CMAM protocol group reached half of that target. As far as we know, this is the first study in west Africa to evaluate coverage of MAM treatment, if we decentralize the treatment at health post. An increase of coverage has been documented by other studies (27, 28, 45–47, among others) that analysed the inclusion of CHWs as SAM treatment providers outside the health centres by making treatment more accessible to communities thus eliminating possible economic barriers. Although we expect a similar effect for MAM treatment in both groups, there are currently no available studies on MAM treatment coverage that include health posts that can be used as comparison. The difference that we have found, may be attributed to the number and spatial distribution of the health posts in each health area. A geospatial study in Niger found that the geographic distribution of community health posts was inefficient, and only 22.1% of the population had access to a treatment site within a 60-min walk (48). In addition, the same study showed that the integration of CHWs in the 7,741 health posts in the country could increase coverage from 41.5% to 82.9%. As our study was not a randomized controlled trial, we cannot ensure that the distribution of the health post was the same in both arms, and this can contribute to the increase in coverage in just the intervention group. This finding could be also due to the protocol used. The control group under the standard protocol used RUSF for MAM, during the study an irregular supply of RUSF for MAM treatment programmes was noted. Management of acute malnutrition with the simplified protocol is easier to apply, could reduce the workload for health service providers, could decrease the waiting time for families, and therefore increase the number of children that can be treated.

In terms of treatment effectiveness, both protocols presented outcomes that exceeded the minimum recommendations for a humanitarian response (>75% recovery, <10% death and < 15% default) (44). The group treated with simplified protocol showed very high recovery rates (99.6%), while the group treated with the CMAM protocol experienced a significant drop to 79.6% recovery due to: (1) a greater number of discharge errors (6.1%), probably due to the fact that a more complex discharge criterion could increase the probability of committing errors and (2) a therapeutic food stock break event, which may have had an effect on increasing non-response (9.9%) and defaulting (4.4%) cases. All this together can be associated with the lower recovery values observed in the CMAM protocol group. Conversely, the ComPAS study by Bailey et al. (30) conducted in South Sudan and Kenya, showed non-significant differences in MAM cure rates between the standard and the same simplified protocol, presenting 85.1% (773/908) and 86.4% (860/995) recovery rates, respectively. Another study by Daures et al. (31) tested a different simplified protocol called OptiMA which consisted of the provision of a reduced RUTF dose based on three MUAC ranges (175 kcal/kg/day with MUAC<115 mm, 125 kcal/kg/day with MUAC 115-120 mm and 75 kcal/kg/day with MUAC 120-124 mm). Comparing like for like MUAC ranges, the OptiMA study presented similar recovery rates to our study (MUAC 115-124 mm: 89.48%, *n* = 3699/4134).

The same simplified protocol as the one used in the present study was tested in a large cohort in Mali which included CHWs as treatment providers in the health posts and recorded a global cure rate for MAM cases of 95.2% (17,220/18,090) (41). In the present study, time to recovery in children treated with the simplified protocol was significantly shorter (28 vs. 42 days with the CMAM protocol). This finding is very consistent with the large Malian cohort reported by Kangas et al. (41), which also recorded a time to recovery of 28 days for MAM cases treated under the same simplified protocol. Some differences have also been found between protocols in terms of consumption of ready-to-use food sachets (60 RUSF sachets with the CMAM protocol vs. 28 RUTF sachets with the simplified protocol) but these values are not directly comparable due to the differences in product composition and costs. The RUTF sachets consumed in the simplified protocol was lower than the 42 sachets recorded in the study by Kangas et al. (41). These differences could be explained by the fact that an extra ration of 7 sachets of RUTF was given to each child at discharge.

Despite a recent meta-analysis on MAM treatment (49) concluding that RUTF and RUSF showed little or no difference in recovery rate (risk ratio: 1.02 [0.98–1.05]), the influence on recovery could be indirect since RUTF may lead to an increase in daily weight gain when compared to RUSF (mean difference of 0.2 g/kg/day; 95% CI: 0.08 to 0.32). If we look at anthropometric gain presented in this study, the group with the simplified protocol far exceeded the CMAM protocol group in both anthropometric indicators (weight: 4.46 vs. 3.08 g/Kg/day; MUAC: 0.34 vs. 0.19 mm/day). These results are also consistent with those found in the Malian cohort (41) which showed a weight gain of 4.8 g/Kg/day and MUAC gain of 0.3 mm/day. In our study, the simplified protocol has been implemented by nurses and CHWs in formal health centres and decentralized health posts. Looking at the effectiveness of the simplified protocol in relation to the treatment provider within the intervention group, we found no differences between CHWs and nurses in terms of cure rate (99.5% vs. 99.6%). In the Malian cohort, the CHWs reached a slightly lower cure ratio of 95.6% (41).

The present study highlights the relevance of decentralizing treatment by involving less skilled CHWs alongside a simplified protocol to optimise effectiveness. Our results showed that children treated by CHWs had lower time to recovery than those treated by health staff (21 vs. 28 days), lower RUTF consumption (28 vs. 35 sachets), higher daily weight gain (6.57 vs. 3.57 g/Kg/day) and MUAC gain (0.46 vs. 0.30 mm/day). This improvement appeared to be gradual as presented in the first dimension of the principal components analysis showing how the simplified protocol resulted in improved outcomes, with the cloud point shifting to the left, which improved even further when including CHWs with a further shift to the left. However, although the observed pattern appears very clear, the complexity surrounding acute malnutrition must not be forgotten, which is also evident in the graph through the dispersed positions of some children.

Some studies on SAM treatment have reported improved outcomes achieved by CHWs could be related to their ability to treat malnutrition and concurrent infections in an integrated manner, thereby reducing the burden of comorbidities that hinder proper recovery. This could be explained by the increased workload of the health staff which limits the time spent diagnosing these pathologies during treatment of acute malnutrition (26, 27). A review on relapse and mortality after discharge (50) highlighted the importance of continuity of care for moderately malnourished cases through integrated and decentralized programmes implemented at the health posts closest to the high-risk populations like those included in the present study.

The multivariate Cox regression model identified treatment protocol, provider and MUAC at admission as factors significantly affecting the probability of recovery over time. The study by Gebremichael (51) also found that MUAC at admission was a significant determinant of recovery. This is because a higher anthropometric status at admission increases the probability of a successful recovery (26, 50). However, our multivariate model found that treatment protocol and provider are more influential that anthropometric status, in this case MUAC, in determining recovery due to the higher adjusted HRs (3.3 and 1.8 respectively). Therefore, treating MAM cases with a simplified protocol that uses RUTF and CHWs improves treatment outcomes. A recent study also concluded that treating high-risk MAM cases with RUTF increased their short-term recovery in terms of greater MUAC and weight gain (20).

The results of our linear regression models reveal that the recovery of acute malnutrition is a complex multifactorial process showed by the *R*^2^ values obtained in our models (around 0.2–0.5) which are relatively low, similar to the results obtained by Maust et al. (34), which had *R*^2^ falling around 0.15–0.5. This suggests that the explanatory variables considered in our four models are not capable of providing a very accurate prediction of the treatment outcomes in terms of time to recovery, consumption of sachets, daily weight and MUAC gain. One of the key results of this study, which aligns with the findings provided by other authors, is that children who receive an integrated treatment, meaning MUAC for diagnosis and RUTF for SAM and MAM treatment, recover faster and with greater gains in MUAC and WHZ. Furthermore, based on our study, we can say that CHWs as treatment providers contribute further improve these outcomes. Identifying other factors influencing recovery and taking them into account in programmatic designs could help improve effectiveness of treatment programmes. A recent study by Rashid et al. (52) identified higher age of the child, higher MUAC at admission, receiving deworming treatment, time taken to access services from the nearby health post (60 min or less) and use of ready-to-use supplementary food as positive predictors of time to recovery from MAM.

The results obtained in the intervention group provide evidence in favour of using a simplified protocol for the treatment of MAM. Moreover, including CHWs as treatment providers, as opposed to relying solely on specialised healthcare personnel, could significantly improve effectiveness of the treatment protocol. In addition, early treatment of malnutrition is more effective, poses less risk to the child, and is less expensive. The cost-effectiveness study of the present research concluded that the cost of treating a MAM child with the CMAM protocol was USD 165.2 (95% CI: 151.7; 179.3), whereas when using the simplified protocol treatment cost was USD 96.5 (95% CI: 87.3; 100.3) (53). Since the mid-nineties’ authors have been highlighting the importance of adequately treating MAM: “there is no question the most severely malnourished children suffer the most, but they may not be contributing to most of the suffering” (54).

### 4.1 Limitations

There are some caveats that should be taken into account. This study is not a randomized controlled trial, so the results cannot be extrapolated to other contexts and the probability of residual confounding is increased. Another possible limitation is the difference in sample sizes between groups, whereby there was a higher incidence of cases in the intervention area. However, this fact actually reflects the field situation, where the worst socioeconomic conditions are recorded, with a higher population of 47,198 habitants vs. a population of 26,176 habitants in the intervention and control groups, respectively. Nevertheless, the statistical tests used are robust about this type of sample imbalance and the results show that the simplification of the protocol is effective even with worse baseline conditions. In this sense, due to logistical reasons, the socioeconomic assessment could not be carried out on all the treated participants, which was corrected by taking a random sample of them. The coverage surveys were carried out in different time periods, just 8 months later after the start of the project. The SQUEAC methodology suggests that this kind of survey can be implemented in periods of a minimum of 4 months after the new intervention is launched for monitoring the effect. One of the factors that may have a negative effect on treatment coverage is the overburdening of health facilities and worsening of access to treatment sites due to flooding, which means not all children in need can reach them. In our case, the period of the highest prevalence of the disease was when the final survey was conducted, and, even with this situation, an increase in treatment coverage was shown in the intervention group when we decentralized treatment to the community level.

## 5 Conclusion

Implementing a simplified protocol for the treatment of MAM based on MUAC as the sole criterion for admission and discharge, a fixed daily dose of one RUTF and involving CHWs as treatment providers could significantly improve the effectiveness and coverage of treatment programmes compared to standard protocols. The simplification of the protocol would facilitate training of personnel and reduce errors due to its easier implementation. In addition, eliminating the requirement to use two different products to treat MAM and SAM cases would simplify the logistics of interventions and align with the intention of treating acute malnutrition continuously and as a whole condition, which is expected to have a positive impact on reducing the therapeutic food stock break events and consequently in increasing treatment coverage since more children will be able to follow the treatment adequately. Furthermore, decentralizing treatment outside of the health centres could be particularly important in emergency settings where access is hindered by multiple barriers. The challenge would be to ensure chain supply closer to the families at the health post level.

Children affected by MAM, not only are very vulnerable due to their condition but also risk deteriorating into SAM if they do not receive timely and adequate treatment. Providing appropriate care to MAM children should be recognised as an important public health issue and prioritised in the future. More research is needed to test this simplified protocol on a larger scale in different contexts to understand its effectiveness in terms of recovery over time and avoid relapses linked to this approach.

## Data availability statement

The original contributions presented in the study are included in the article/supplementary material, further inquiries can be directed to the corresponding author.

## Ethics statement

The studies involving humans were approved by National Health Research Ethics Committee of Niger. The studies were conducted in accordance with the local legislation and institutional requirements. Written informed consent for participation in this study was provided by the participants’ legal guardians/next of kin.

## Author contributions

LS-M: Data curation, Formal analysis, Validation, Visualization, Writing – original draft. PC-C: Conceptualization, Funding acquisition, Methodology, Supervision, Validation, Writing – review & editing. AG: Project administration, Supervision, Validation, Writing – review & editing. AD: Project administration, Supervision, Validation, Writing – review & editing. AS: Supervision, Validation, Writing – review & editing. NO: Supervision, Validation, Writing – review & editing. RL: Supervision, Validation, Writing – review & editing. FT: Funding acquisition, Project administration, Validation, Writing – review & editing. AV: Funding acquisition, Validation, Writing – review & editing. CH: Validation, Visualization, Writing – review & editing. NL-E: Conceptualization, Data curation, Formal analysis, Investigation, Methodology, Supervision, Validation, Writing – review & editing.

## References

[1] UNICEF, WHO, World Bank Group. Levels and trends in child malnutrition (2021). Available at: https://www.who.int/publications/i/item/9789240025257

[2] Institut National de la Statistique (INS). Enquête nutritionnelle et de mortalité retrospective au Niger. (2021). Available at: https://fscluster.org/niger/document/ins-enquete-nutritionnelle-et-de

[3] de Onis M Borghi E Arimond M Webb P Croft T Saha K. Prevalence thresholds for wasting, overweight and stunting in children under 5 years. Public Health Nutr. (2019) 22:175–9. doi: 10.1017/S1368980018002434, PMID: 30296964 PMC6390397

[4] Food and Agriculture Organization (FAO). The Niger: Response overview (2022). Available at: 10.4060/cc0587en

[5] World Food Programme (WFP), Rapport Sommaire du Coût de la Faim en Afrique (COHA): L’Incidence Sociale et Economique de la Malnutrition Chez L’enfant au Niger. (2018). Available at: https://reliefweb.int/report/niger/le-co-t-de-la-faim-en-afrique-l-incidence-sociale-et-conomique-de-la-malnutrition-chez

[6] United Nations Children’s Fund (UNICEF). Severe Acute Malnutrition. (2018). Available at: https://www.unicef.org/nutrition/index_sam.html

[7] Black RE Allen LH Bhutta ZA Caulfield LE de Onis M Ezzati M. Maternal and child undernutrition: global and regional exposures and health consequences. Lancet. (2008) 371:243–60. doi: 10.1016/S0140-6736(07)61690-0, PMID: 18207566

[8] Black RE Victora CG Walker SP Bhutta ZA Christian P de Onis M. Maternal and child undernutrition and overweight in low-income and middle-income countries. Lancet. (2013) 382:427–51. doi: 10.1016/S0140-6736(13)60937-X 23746772

[9] Olofin I McDonald CM Ezzati M Flaxman S Black RE Fawzi WW. Associations of suboptimal growth with all-cause and cause-specific mortality in children under five years: a pooled analysis of ten prospective studies. PLoS One. (2013) 8:e64636. doi: 10.1371/journal.pone.0064636, PMID: 23734210 PMC3667136

[10] Stobaugh HC Bollinger LB Adams SE Crocker AH Grise JB Kennedy JA. Effect of a package of health and nutrition services on sustained recovery in children after moderate acute malnutrition and factors related to sustaining recovery: a cluster-randomized trial. Am J Clin Nutr. (2017) 106:657–66. doi: 10.3945/ajcn.116.149799, PMID: 28615258 PMC6482975

[11] World Health Organization (WHO). Supplementary foods for the Management of Moderate Acute Malnutrition in infants and children 6–59 months of age. (2012). Available at: https://apps.who.int/iris/handle/10665/75836

[12] World Health Organization (WHO). Guideline: Updates on the Management of Severe Acute Malnutrition in infants and children. Geneva: WHO. (2013). Available at: https://www.who.int/publications/i/item/9789241506328 (Accessed December 13, 2022) 24649519

[13] Dalglish SL Badou MS Sirat A Abdullahi O Adalbert MFE Biotteau M. Combined protocol for severe and moderate acute malnutrition in emergencies: stakeholders perspectives in four countries. Matern Child Nutr. (2020) 16:e12920. doi: 10.1111/mcn.12920, PMID: 31773867 PMC7083443

[14] Bhutta ZA Ahmed T Black RE Cousens S Dewey K Giugliani E. What works? Interventions for maternal and child undernutrition and survival. Lancet. (2008) 371:417–40. doi: 10.1016/S0140-6736(07)61693-6 18206226

[15] Lenters LM Wazny K Webb P Ahmed T Bhutta ZA. Treatment of severe and moderate acute malnutrition in low-and middle-income settings: a systematic review, Meta-analysis and Delphi process. BMC Public Health. (2013) 13:S23. doi: 10.1186/1471-2458-13-S3-S23, PMID: 24564235 PMC3847503

[16] World Health Organization (WHO). Assessing and managing children at primary health-care facilities to prevent overweight and obesity in the context of the double burden of malnutrition. Updates for the integrated management of childhood illness (IMCI) – guideline. (2017). Available at: https://www.who.int/nutrition/publications/guidelines/children-primaryhealthcare-obesity-dbm/en/29578661

[17] Lelijveld N Beedle A Farhikhtah A Elrayah EE Bourdaire J Aburto N. Systematic review of the treatment of moderate acute malnutrition using food products. Matern Child Nutr. (2020) 16:e12898. doi: 10.1111/mcn.12898, PMID: 31667981 PMC7038867

[18] Das JK Salam RA Saeed M Kazmi FA Bhutta ZA. Effectiveness of interventions for managing acute malnutrition in children under five years of age in low-income and middle-income countries: a systematic review and Meta-analysis. Nutrients. (2020) 12:116. doi: 10.3390/nu12010116, PMID: 31906272 PMC7019612

[19] Gluning I Kerac M Bailey J Bander A Opondo C. The management of moderate acute malnutrition in children aged 6-59 months in low-and middle-income countries: a systematic review and meta-analysis. Trans R Soc Trop Med Hyg. (2021) 115:1317–29. doi: 10.1093/trstmh/trab137, PMID: 34535798

[20] Lelijveld N Godbout C Krietemeyer D Los A Wegner D Hendrixson DT. Treating high-risk moderate acute malnutrition using therapeutic food compared with nutrition counseling (hi-MAM study): a cluster-randomized controlled trial. Am J Clin Nutr. (2021) 114:955–64. doi: 10.1093/ajcn/nqab137, PMID: 33963734 PMC8921644

[21] Rogers E Myatt M Woodhead S Guerrero S Alvarez JL. Coverage of community-based Management of Severe Acute Malnutrition Programmes in twenty-one countries, 2012-2013. PLoS One. (2015) 10:e0128666. doi: 10.1371/journal.pone.0128666, PMID: 26042827 PMC4456359

[22] James P Sadler K Wondafrash M Argaw A Luo H Geleta B. Children with moderate acute malnutrition with no access to supplementary feeding Programmes experience high rates of deterioration and no improvement: results from a prospective cohort study in rural Ethiopia. PLoS One. (2016) 11:e0153530. doi: 10.1371/journal.pone.0153530, PMID: 27100177 PMC4839581

[23] United Nations Children’s Fund (UNICEF) Treatment of wasting using simplified approaches—A rapid evidence review. (2020). Available at: https://www.unicef.org/media/97006/file

[24] Alé FGB Phelan KPQ Issa H Defourny I Le Duc G Harczi G. Mothers screening for malnutrition by mid-upper arm circumference is non-inferior to community health workers: results from a large-scale pragmatic trial in rural Niger. Arch Public Health. (2016) 74:38. doi: 10.1186/s13690-016-0149-5, PMID: 27602207 PMC5011948

[25] Bliss J Lelijveld N Briend A Kerac M Manary M McGrath M. Use of mid-upper arm circumference by novel community platforms to detect, diagnose, and treat severe acute malnutrition in children: a systematic review. Glob Health Sci Pract. (2018) 6:552–64. doi: 10.9745/GHSP-D-18-00105, PMID: 30185435 PMC6172115

[26] López-Ejeda N Charle-Cuellar P Alé FGB Álvarez JL Vargas A Guerrero S. Bringing severe acute malnutrition treatment close to households through community health workers can lead to early admissions and improved discharge outcomes. PLoS One. (2020) 15:e0227939. doi: 10.1371/journal.pone.0227939, PMID: 32023265 PMC7001926

[27] Dougnon AO Charle-Cuéllar P Toure F Gado AA Sanoussi A Lazoumar RH. Impact of integration of severe acute malnutrition treatment in primary health care provided by community health Workers in Rural Niger. Nutrients. (2021) 13:4067. doi: 10.3390/un13114067, PMID: 34836322 PMC8625976

[28] Charle-Cuéllar P Lopez-Ejeda N Souleymane HT Yacouba D Diagana M Dougnon AO. Effectiveness and coverage of treatment for severe acute malnutrition delivered by community health Workers in the Guidimakha Region, Mauritania. Children. (2021) 8:1132. doi: 10.3390/children8121132, PMID: 34943328 PMC8700149

[29] Charle-Cuéllar P López-Ejeda N Traore M Coulibaly AB Landouré A Diawara F. Impact of different levels of supervision on the recovery of severely malnourished children treated by community health Workers in Mali. Nutrients. (2021) 13:367. doi: 10.3390/nu13020367, PMID: 33530333 PMC7911749

[30] Bailey J Opondo C Lelijveld N Marron B Onyo P Musyoki EN. A simplified, combined protocol versus standard treatment for acute malnutrition in children 6-59 months (ComPAS trial): a cluster-randomized controlled non-inferiority trial in Kenya and South Sudan. PLoS Med. (2020) 17:e1003192. doi: 10.1371/journal.pmed.1003192, PMID: 32645109 PMC7347103

[31] Daures M Phelan K Issoufou M Kouanda S Sawadogo O Issaley K. New approach to simplifying and optimising acute malnutrition treatment in children aged 6-59 months: the OptiMA single-arm proof-of-concept trial in Burkina Faso. Br J Nutr. (2020) 123:756–67. doi: 10.1017/S0007114519003258, PMID: 31818335 PMC7054246

[32] Lelijveld N Musyoki E Adongo SW Mayberry A Wells JC Opondo C. Relapse and post-discharge body composition of children treated for acute malnutrition using a simplified, combined protocol: a nested cohort from the ComPAS RCT. PLoS One. (2021) 16:e0245477. doi: 10.1371/journal.pone.0245477, PMID: 33534818 PMC7857614

[33] Cazes C Phelan K Hubert V Boubacar H Bozama LI Sakubu GT. Simplifying and optimising the management of uncomplicated acute malnutrition in children aged 6-59 months in the Democratic Republic of the Congo (OptiMA-DRC): a non-inferiority, randomised controlled trial. Lancet Glob Health. (2022) 10:e510–20. doi: 10.1016/S2214-109X(22)00041-9, PMID: 35303461

[34] Maust A Koroma AS Abla C Molokwu N Ryan KN Singh L. Severe and moderate acute malnutrition can be successfully managed with an integrated protocol in Sierra Leone. J Nutr. (2015) 145:2604–9. doi: 10.3945/jn.115.214957, PMID: 26423737

[35] World Health Organization (WHO). (2006), Child growth standards, Available at: https://www.who.int/publications/i/item/924154693X

[36] Nutriset. Acute malnutrition prevention and treatment products: Plumpy-nut and Plumpy-sup. (2018). Available at: https://www.nutriset.fr/en/products

[37] World Food Programme (WFP). Vulnerability analysis and mapping. Food consumption analysis. Calculation and 523 use of the food consumption score in food security analysis (2008). Available at: https://documents.wfp.org/stellent/groups/public/documents/manual_guide_proced/wfp197216.pdf

[38] Myatt M Guevarra E Fieschi L Norris A Guerrero S Schofield L. Semi-quantitative evaluation of access and coverage (SQUEAC) / simplified lot quality assurance sampling evaluation of access and coverage (SLEAC) technical reference. (2012). Available at: https://www.fantaproject.org/monitoring-and-evaluation/squeac-sleac

[39] R Core Team. R: A language and environment for statistical computing. Vienna, Austria: R Foundation for Statistical Computing (2022).

[40] Rosner B. Fundamentals of biostatistics. 7th ed. Boston, MA: Brooks/Cole (2011). 381 p.

[41] Kangas ST Marron B Tausanovitch Z Radin E Andrianarisoa J Dembele S. Effectiveness of acute malnutrition treatment at health center and community levels with a simplified, combined protocol in Mali: an observational cohort study. Nutrients. (2022) 14:4923. doi: 10.3390/nu14224923, PMID: 36432609 PMC9699530

[42] Bradburn MJ Clark TG Love SB Altman DG. Survival analysis part II: multivariate data analysis – an introduction to concepts and methods. Br J Cancer. (2003) 89:431–6. doi: 10.1038/sj.bjc.6601119, PMID: 12888808 PMC2394368

[43] Tsegaye A Lencha B Kumsa K. Predictors of time to recovery from uncomplicated severe acute malnutrition among 6-59 months children treated in outpatient treatment in health posts of Nagele Arsi district: a retrospective cohort study. BMC Pediatr. (2022) 22:712. doi: 10.1186/s12887-022-03767-4, PMID: 36514008 PMC9746122

[44] The sphere project. Minimum standards in food security and nutrition. In: Humanitarian charter and minimum standards in humanitarian response. Sphere Handbook. (2018). Available at: https://spherestandards.org/es/el-manual/

[45] Álvarez-Morán JL Alé FGB Charle P Sessions N Doumbia S Guerrero S. The effectiveness of treatment for severe acute malnutrition (SAM) delivered by community HealthWorkers compared to a traditional facility based model. BMC Health Serv Res. (2018) 18:207. doi: 10.1186/s12913-018-2987-z, PMID: 29580238 PMC5870488

[46] López-Ejeda N Charle-Cuellar P Vargas A Guerrero S. Can community health workers manage uncomplicated severe acute malnutrition? A review of operational experiences in delivering severe acute malnutrition treatment through community health platforms. Matern Child Nutr. (2018) 15:e12719. doi: 10.1111/mcn.12719, PMID: 30315743 PMC6587873

[47] Wilunda C Mumba FG Putoto G Maya G Musa E Lorusso V. Effectiveness of screening and treatment of children with severe acute malnutrition by community health workers in Simiyu region, Tanzania: a quasi-experimental pilot study. Sci Rep. (2021) 11:2342. doi: 10.1038/s41598-021-81811-6, PMID: 33504865 PMC7840757

[48] Oliphant NP Ray N Bensaid K Ouedraogo A Gali AY Habi O. Optimising geographical accessibility to primary health care: a geospatial analysis of community health posts and community health workers in Niger. BMJ Glob Health. (2021) 6:e005238. doi: 10.1136/bmjgh-2021-005238, PMID: 34099482 PMC8186743

[49] Cichon B Das JK Salam RA Padhani ZA Stobaugh HC Mughal M. Effectiveness of dietary Management for Moderate Wasting among children > 6 months of age-a systematic review and Meta-analysis exploring different types, quantities, and durations. Nutrients. (2023) 15:1076. doi: 10.3390/nu15051076, PMID: 36904076 PMC10005276

[50] Stobaugh HC Mayberry A McGrath M Bahwere P Zagre NM Manary MJ. Relapse after severe acute malnutrition: a systematic literature review and secondary data analysis. Mat Child Nutr. (2019) 15:e12702. doi: 10.1111/mcn.12702, PMID: 30246929 PMC6587999

[51] Gebremichael DY. Predictors of nutritional recovery time and survival status among children with severe acute malnutrition who have been managed in therapeutic feeding centers, southern Ethiopia: retrospective cohort study. BMC Public Health. (2015) 15:1267. doi: 10.1186/s12889-015-2593-5, PMID: 26689192 PMC4687080

[52] Rashid MY Kebira JY Oljira L Dheresa M. Time to recovery from moderate acute malnutrition and its predictors among children 6–59 months of age enrolled in targeted supplementary feeding program in Darolebu District, eastern Ethiopia: a retrospective cohort study. Front Public Health. (2022) 10:914837. doi: 10.3389/fpubh.2022.914837, PMID: 35910899 PMC9330372

[53] Cichon B Lopez-Ejeda N Charle-Cuellar P Hamissou IA Karim AAA Aton C. Cost of acute malnutrition treatment using a simplified or standard protocol in Diffa. Niger Nutrients. (2023) 15:3833. doi: 10.3390/nu15173833, PMID: 37686865 PMC10490076

[54] Yip R Scanlon K. The burden of malnutrition: a population perspective. J Nutr. (1994) 124:2043S–6S. doi: 10.1093/jn/124.suppl_10.2043S 7931715

